# FoxO3a regulated by miR-150-5p promotes the pyroptosis of macrophages in atherosclerosis

**DOI:** 10.1371/journal.pone.0327075

**Published:** 2025-10-17

**Authors:** Ping-yu Cai, Mei-mei Li, Shu-han Chen, Yan-li Zheng, Jun Li, De-hong Huang, Jing-ru Du, Hui-li Lin

**Affiliations:** Department of Cardiology, the Second Affiliated Hospital of Fujian Medical University, Quanzhou, Fujian, China; Noorda College of Osteopathic Medicine, UNITED STATES OF AMERICA

## Abstract

**Background:**

This study uses tissue exosome analysis to explore the role of miR-150-5p and its downstream genes in atherosclerosis (AS), an area where the functional mechanisms and pathophysiological significance of exosomal miR-150-5p remain poorly understood.

**Methods:**

Exosomes from AS mouse vascular tissue were analyzed to identify miR-150-5p target genes. Dual luciferase assays validated miRNA-target interactions, while RT-qPCR and Western blot assessed FoxO3a expression. RNA interference studies determined FoxO3a’s role in pyroptosis. In vivo efficacy of the miR-150-5p inhibitor was evaluated using HE, Masson staining, and immunofluorescence.

**Results:**

In AS tissue exosomes, miR-150-5p levels increased whereas FoxO3a levels decreased. miR-150-5p regulated FoxO3a, enhancing macrophage pyroptosis. The miR-150-5p inhibitor reduced ox-LDL-induced RAW264.7 injury and pyroptosis by improving cell viability, decreasing LDH levels, and downregulating pyroptosis related proteins (Caspase-1, NLRP3, GSDMD-N). FoxO3a knockdown weakened the inhibitor’s effects on NLRP3/GSDMD-mediated pyroptosis. In Apoe^−/−^ mice, the inhibitor upregulated FoxO3a/ARC and suppressed pyroptosis signaling.

**Conclusion:**

This study advances understanding of miR-150-5p-mediated pyroptosis and highlights the potential of miR-150-5p inhibitors in combating AS.

## Introduction

Atherosclerosis (AS) is a major contributor to cardiovascular disease, leading to millions of deaths globally each year [1,2]. In China, cardiovascular disease cases and related deaths are rising sharply, exceeding gross domestic product (GDP) growth and straining the healthcare system [3]. Thus, insights into AS’s molecular mechanisms are essential for preventing and treating these diseases.

AS pathology involves endothelial dysfunction, LDL oxidation, immune cell activation, and cytokines, leading to VSMC migration, foam cell formation, and cell death [4]. Pyroptosis releases inflammatory mediators like IL-1β and IL-18, promoting AS inflammation [5,6]. Emerging evidence indicates that pyroptosis promotes plaque instability and atherosclerosis progression by exacerbating lipid accumulation and inflammatory responses, which can ultimately lead to plaque rupture and acute cardiovascular events [7–9]. Macrophage cytokines (e.g., TNF-α, IL-6) intensify local inflammation and pyroptosis through pathways such as NF-κB signaling and caspase-1 activation, thereby contributing to plaque destabilization. Susceptible plaques result from macrophage changes and lipid-activated pyroptosis, triggering cytokine release and fragility. Understanding macrophage pyroptosis in AS could reveal therapeutic targets and clarify its pathogenesis.

Forkhead box O (FoxO) protein is crucial for cellular homeostasis, regulating energy production, oxidative stress resistance, and cell survival [10]. FoxO3 specifically inhibits pyroptosis by modulating inflammatory responses [11]. Studies show miR-30d and miR-155 targeting FoxO3a regulate myocardial cell injury and cardiomyocyte pyroptosis, respectively [12–14]. However, both the specific role of FoxO3a in modulating pyroptosis during AS and its underlying molecular mechanism remain poorly understood.

Exosomes are extracellular particles released by a variety of cell types that contain non-coding RNAs like circRNA and miRNA. Of these, miRNA is a member of a class of tiny non-coding RNAs that are about 20–22 nucleotides long and have the ability to control post-transcriptional levels of gene expression [15]. MiRNA is directly linked to cardiovascular disease and has a significant regulatory role in cellular processes like differentiation, apoptosis, and proliferation [16–19]. They influence various vascular related cells, contributing to atherosclerotic plaque development. For example, Bian et al. [20] found that miR-150-5p may stabilize atherosclerotic plaques and regulate the proliferation and migration of ox-LDL-stimulated AS cells by targeting STAT1. Another study showed that ZFAS1 promotes Notch3 activation and facilitates oxLDL-induced endothelial mesenchymal transition by acting as a ceRNA for miR-150-5p [21]. miR-150-5p, a newly discovered death-related miRNA, is predicted to be a key regulator of FoxO3a. Studies suggest that miR-150-5p promotes pyroptosis in cells like cardiomyocytes [22] and hepatocytes [23]. However, its role in AS macrophage pyroptosis is not yet fully understood.

Apoptosis inhibitors containing caspase recruitment domains (ARC) are anti-apoptotic proteins that counteract both intrinsic and extrinsic cell death pathways. They are FoxO3a transcriptional targets [24]. Research indicates that miR-30d inhibits ARC and targets FoxO3a to induce cardiomyocyte pyroptosis [25]. Melatonin, known for its protective effects, increases ARC levels and inhibits apoptosis in H9c2 cells, while ARC silencing blocks melatonin’s pyroptosis-inducing activity [26]. Therefore, the FoxO3a/ARC pathway is crucial in regulating cell apoptosis.

The function of miR-150-5p in exosomes produced from vascular tissue during AS was examined in this work. We discovered FoxO3a-ARC to be a critical downstream receptor of miR-150-5p when comparing the normal and AS groups, providing information for targeted AS treatments.

## Materials and methods

### Experimental animals

This study, approved by the Animal Care Committee of the Second Affiliated Hospital of Fujian Medical University, followed NIH guidelines (No. 2023–379). Apoe knockout (Apoe^−/−^) C57BL/6J male mice (6–7 weeks, 25 ± 5g) were sourced from SLACK Laboratory Animal Company (Shanghai). Maintained in a specific pathogen-free facility, mice were fed a high-fat diet (40% fat, 40% carbohydrates, and 20% protein) for 12 weeks to induce plaque formation, with euthanization occurring up to 12 weeks post-intervention. Animals were fasted for 12 hours and euthanized with pentobarbital sodium (150 mg/kg, i.p.). Surgery utilized isoflurane anesthesia (3% induction, 1.5% maintenance), with postoperative buprenorphine (0.05 mg/kg, s.c., every 8 h for 48 h) for analgesia. Soft bedding and environmental controls were provided to minimize discomfort.

### Separation of exosomes from tissue

Using differential ultracentrifugation, exosomes were separated. Following a PBS rinse, tissue samples were sliced into 1 mm³ pieces and digested for an hour at 37 °C using 0.2% type I collagenase. Exosomes were isolated from the homogenate of digested whole vascular segments, without micro-dissection of specific sub-regions. The exosomal population thus represents a mixture derived from all plaque areas and cell types within the atherosclerotic tissue. 10% exosome-free serum in DMEM was used to halt digestion. After centrifugation at 2000 × g for 15 minutes and then again at 4 °C for 30 minutes, the supernatant was recovered. Centrifugation at 10,000 × g for 45 minutes at 4 °C was used for separating larger vesicles. A 0.45 μm membrane was used to filter the supernatant, which was then ultracentrifuged at 100,000 × g for 70 minutes at 4 °C. The pellet was centrifuged once again at 100,000 × g for 70 minutes at 4 °C after being resuspended in 10 mL of PBS that had been chilled beforehand. One hundred microliters of chilled PBS were used to resuspend the last exosome particle.

### Transmission electron microscopy

A volume of 10 μL was generated by diluting the exosomes in a 1:2 ratio. Filter paper was used to absorb the foating liquid after the sample had been precipitated by pouring 10 μL to a copper grid for one minute. Filter paper was used to extract the foating liquid after uranium hydroxide (10 μL) was introduced dropwise to the metal mesh to precipitate for one minute. At room temperature, the samples were left to dry. The imaging data (Hitachi, HT-7700) were acquired using a 100 kV electron microscope.

### Particle size analysis

To ascertain the exosomes’ size distribution and concentration, a nano flow cytometer (NanoFCM, N30E) was employed. To achieve a total volume of 30 μL, the exosomes were diluted at a 1:6 ratio. The exosome sample was added after the successful finishing of the regular instrument performance test. The need for gradient dilution to prevent the sample from obstructing the injection needle was taken into concern. Following sample its conclusion, data on the concentration and size of exosomes identified by the device can be extracted.

### Fluorescent labelling and nanofuid detection

The Flow NanoAnalyzer (N30E, Life Internet Technology Co., Ltd.) was used to perform nanoflow cytometry. Following instrument calibration, exosome samples (10 μL) were diluted to 30 μL and loaded. Exosome size and concentration were provided by the results. After diluting 30 μL of the exosome sample to 120 μL, 20 μL of fluorescent antibodies (CD9, CD63, and CD81) were added, and the mixture was incubated for 30 minutes at 37°C. After centrifuging the mixture for 70 minutes at 4°C at 110,000 × g, it was rinsed with PBS and resuspended in 50 μL of PBS. The device produced protein marker results following retesting.

### High‑throughput sequencing and bioinformatics analysis

After determining the concentration of exosomal RNA, NR1 clipping matched diluent was added to the RNA stock solution to dilute it. The machine was then used to test the diluted samples. The following criteria were used to filter low-quality data: reads with adapters in order reads with N ratios >10%, reads with all A bases, low-quality reads (Q ≤ 20), and sequences with effective lengths <20 nt. Fastp software was used to check the data for quality control. Following filtering, clean reads were acquired, and STAR software was used to map them to the standard genome (NCBI assembly mRatBN7.2). DESeq2 was used for differential expression analysis, and feature counts were used to estimate and annotate protein-coding genes. Genes getting a significance criterion of p-value <0.05 and |log FC| ≥ 1 were identified to be differentially expressed.

To predict potential miRNA-mRNA interactions, the TarBase (http://microrna.gr/tarbase/) and miRTarbase (https://miRTarBase.cuhk.edu.cn/) databases were used to search for miR-150-5p target genes. The target genes were evaluated using the string database and Cytoscape; functional enrichment analysis was carried out using the analysis website Metascape (https://metascape.org/gp/index.html#/main/step1).

### Quantitative reverse transcription polymerase chain reaction (RT‑qPCR)

Using a centrifugal column technique, total RNA was extracted from tissues or cells, and spectrophotometry was used to assess its purity and concentration. The PrimeScript RT kit was then used to reverse transcribe the RNA into cDNA. Using GAPDH and U6 as endogenous controls, RT-qPCR was carried out three times using particular primers to assess mRNA expression in vascular tissue. A StepOne Plus real-time PCR supplies was used for quantitative expression analysis, and the 2 − ΔΔCt technique was used to calculate relative mRNA expression.

### Western blot analysis

Protease/phosphatase inhibitor-containing RIPA buffer was utilized to lyse tissues or cells. After standardizing the samples, they were centrifuged for 15 minutes at 4°C at 12,000 rpm. Protein samples were transferred to PVDF membranes, electrophoresed on SDS-PAGE gels, and incubated with 5% milk in TBS-T. HRP-conjugated secondary antibodies were incubated for one hour at room temperature following primary antibodies were incubated for an entire night at 4°C. Chemiluminescence reagents were used to visualize Western blotting.

### Cell culture and transfection

miR-150-5p mimics, miR-150-5p inhibitor, and corresponding control, plasmid of FoxO3a overexpression and small interfering RNA targeting FoxO3a (si FoxO3a) were synthesized by Hanheng Biotechnology Co., Ltd. (Shanghai, China). Lipofectamine 3000 (Invitrogen, Carlsbad, CA, USA) was used to transfect cells with plasmid DNA, siRNA, or miRNA inhibitors/mimics. After 24 hours of transfection, cells were collected to determine knockdown efficiency using RT-qPCR.

### CCK‑8 and LDH assays

96-well plates were seeded with RAW264.7. After various treatments, cells were incubated for two hours with a 10% CCK-8 reaction solution. A reader for micro plates (Spectramax i3x, USA) was used to measure optical densities (ODs) at 450 nm. The percentage of the baseline condition was used for assessing the viability.

Using a particular LDH detection kit, the LDH released into the culture medium was measured to assess RAW264.7 injury in accordance with the manufacturer’s recommendations.

### Dual‑luciferase reporter assay

Targeting predicted miR-150-5p binding sites, FoxO3a a wild-type (FoxO3a-WT) and mutant (FoxO3a-MUT) sequences were constructed using the psi-CHECK2 vector. Luciferase reporter vectors were used to co-transfect 293T cells with either miR-NC or miR-150-5p. A dual-luciferase reporter assay kit was used for evaluating luciferase activity after 48 hours, reducing firefly luciferase levels to renilla luciferase values. This validated FoxO3a’s binding connection with miR-150-5p.

### Establishment of animal models

For eight weeks, twenty-four APOE − / − mice were fed a high-fat diet (HFD) containing 40% fat, 40% carbohydrates, and 20% protein. After subsequently, the mice were divided into four groups at random, with six mice in each group. Blinding was applied during outcome assessment to prevent bias: data collection and analysis were performed by investigators unaware of group assignments. (1) Control group (n = 6): usual diet, no treatment. (2) Model group (n = 6): 12 weeks of a high-fat diet. 200 μL of PBS was administered into the tail vein following nine weeks of modeling. (3) NC inhibitor group (n = 6): 200 μL of viral NC inhibitor was administered into the tail vein following nine weeks of modeling. (4) miR-150-5p inhibitor group (n = 6): 200 μL of viral miR-150-5p inhibitor had been given into the tail vein following nine weeks of modeling. The mice had to be killed at the finish of the experiment, and blood samples were taken from the apex of their hearts. The hearts, along with their aortic roots, were then removed transversely. The aortic arches were then gathered, immersed in liquid nitrogen, and placed in a refrigerator set at −80 °C for additional analysis.

### Immunohistochemistry

Tissue slices were treated with 3% hydrogen peroxide to inhibit endogenous peroxidase activity following deaffinity, dehydration, and antigen retrieval. After an hour of blocking with goat serum, they were incubated with the primary antibody against either ARC (1:200; Proteintech) or FoxO3a (1:300; Proteintech) for the entire night at 4°C. Sections were then treated for 60 minutes at room temperature with the proper secondary antibodies. DAB exposure at room temperature and hematoxylin counterstaining were used to visualize immunoreactivity. After that, sections were coverslipped and dehydrated. Image-J software was used to measure the mean optical denseness in three to five arbitrarily fields per slice in order to quantify immunoreactivity.

### Immunofluorescence

Before being fixed using 0.5% Triton X-100 for infiltration, cells were sown on 15 mm confocal culture dishes (NEST) and fixed with formaldehyde for 20 minutes. The cells were treated with specific primary Ab Caspase-1 (1:500; Affinity) following 30 minutes of incubation with 5% donkey serum (Sorabio). After 14 hours at 4°C, the cells were treated for 60 minutes in the dark with donkey anti-rabbit 488 (1:500; Smyrph). After capping the film for 15 minutes with a DAPI-containing antifluorescence quencher, a Nikon confocal microscope was used to take pictures.

### Statistical analysis

Three separate biological studies’ means±standard variations were used to express the data, and GraphPad Prism 9.5.0 was used for the graphs. Two groups were evaluated using the Student’s t-test, and multiple groups were examined using a one-way ANOVA. Statistical significance was set at *p < 0.05, **p < 0.01, ***p < 0.001, ****p < 0.0001.

## Results

### Pyroptosis occurs in the pathogenesis of AS

To assess weight changes, mice weights were measured every 2 weeks throughout the experimental period (S1A and S1B Fig). H&E staining revealed that the vascular wall structure in control group mice was clear, with normal cell morphology and no thrombus formation. In contrast, the model group showed thickened vascular walls, lipid deposition, and significant lumen stenosis (S1C and S1D Fig). After modeling, ELISA was used to analyze serum inflammatory factors, and the levels of IL-1 β and IL-18 were significantly increased in the high-fat diet group of mice (S1E and S1F Fig).

The experimental animals’ protein levels of NLRP3, GSDMD-N, and Caspase-1 were higher than those of the control group, according to Western blot analysis (S1G and S1H Fig). RT-qPCR verified that the mRNA expression of these same markers was also higher (S1I Fig). These findings confirm pyroptosis involvement in AS pathogenesis.

### Expression of miR-150-5p and its downstream pathway in the HFD group

#### Extraction and identifcation of exosomes from vascular tissue.

**Fig 1:**
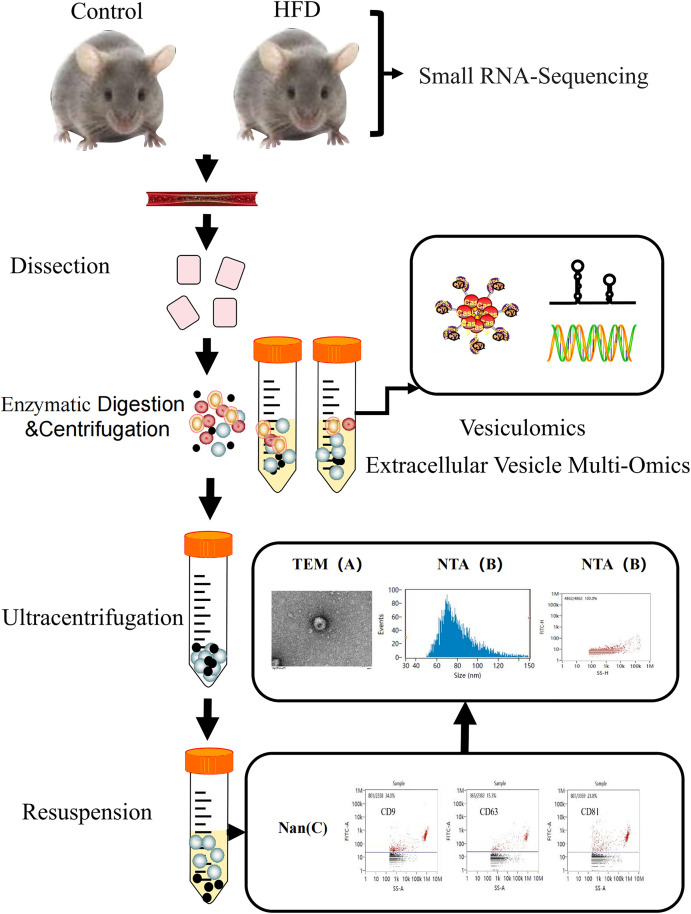
Experimental workflow for isolation and characterization of vascular tissue-derived exosomes. Transcriptomic studies were conducted on vascular tissue samples from the internal carotid artery to the iliac artery in mice. These sample types were subjected to enzymatic hydrolysis and a series of low and high-speed centrifugation treatments. Then, the high-speed supernatant was subjected to ultracentrifugation washing/granular extracellular vesicles (EVs). Use transmission electron microscopy (TEM), nanoparticle tracking analysis (NTA), and nanoflow cytometry (Nan) to identify and classify these EVs.

This article used differential centrifugation to isolate exosomes from mouse vascular tissue. pass through transmission electron microscopy (TEM) observed that two groups of extracellular vesicles were circular and elliptical in shape (Fig 1A), with a cup-shaped structure indicating the presence of a bilayer lipid envelope. Combined with the results of nanoparticle tracking analysis (NTA), it was found that the majority of the extracellular vesicles were concentrated between 60–100 nm in size and morphology (Fig 1B), which is consistent with the size and morphology of the extracellular vesicles. In addition, this study used nanoflow cytometry (nFCM) to detect the characteristic membrane proteins CD9, CD63, and CD81 of the extracellular vesicles, all of which were positive (Fig 1C). These results indicate that the extracellular vesicles we isolated by differential centrifugation are exosomes.

#### Screening and validation of DEGs.

**Fig 2:**
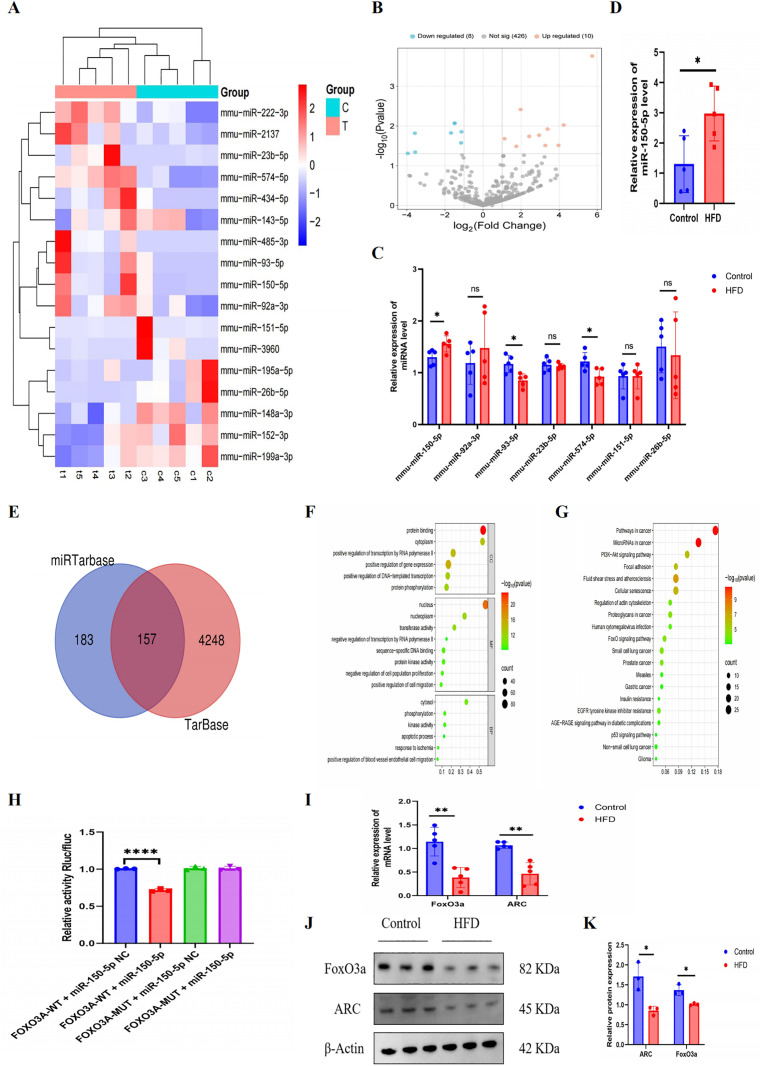
Dysregulation of miR-150-5p and its target FoxO3a in atherosclerosis (AS). (A) Heatmap and (B) volcano plot of differentially expressed miRNAs in exosomes from control versus AS groups. (C) Stem-loop RT-qPCR quantification showing upregulated miR-150-5p and downregulated miR-574-5p/miR-93-5p in AS vascular tissue exosomes. (D) miR-150-5p expression in mouse arterial plaques. (E) Venn diagram of miR-150-5p target genes predicted by miRTarBase and TarBase databases. (F) GO and (G) KEGG pathway enrichment analysis of target genes. (H) Dual-luciferase reporter assay confirming miR-150-5p-FoxO3a binding. (I) RT-qPCR analysis of FoxO3a and ARC mRNA expression in vascular tissues. (J-K) Western blot quantification of FoxO3a and ARC protein levels. Data are described with mean±SD of at least 3 diferent experiments. ns = not signifcant; *P < 0.05; **P < 0.01; ****P < 0.0001 vs. cells in the control group.

Using a threshold of *p* < 0.05 and |log2(fold change)| ≥ 1, 17 differentially expressed miRNAs were identified. Compared with normal vascular tissue exosomes, 10 were upregulated and 7 were downregulated in AS, as shown in clustering and volcano plots (Fig 2A and 2B). Stem loop RT-qPCR confirmed upregulation of miR-150-5p and downregulation of miR-93-5p and miR-574-5p in AS (Fig 2C). Due to the significant upregulation and significant differences in miR-150-5p, it was chosen for further research. RT-qPCR validation in vascular tissue confirmed an increase in miR-150-5p expression in AS (*p* < 0.05, Fig 2D).

#### Identifcation of miR‑150‑5p as potential upstream regulator of FoxO3a.

157 overlapping target genes were found after screening for miR-150-5p target genes using miR-TarBase and TarBase (Fig 2E). Pathways like p53, FoxO, and PI3K Akt were revealed by GO and KEGG enrichment analysis (Fig 2F and 2G). FoxO proteins, especially FoxO3a, are involved in cell death and proliferation, and may regulate pyroptosis [13,27]. Therefore, FoxO3a is considered a candidate target gene for miR-150-5p regulation of macrophage pyroptosis in AS.

Dual-luciferase reporter assays confirmed that miR-150-5p directly binds to the 3’UTR of FoxO3a, as evidenced by the significant decrease in luciferase activity. This result provides direct mechanistic evidence for the transcriptional negative regulation of FoxO3a by miR-150-5p (*p* < 0.05; Fig 2H). Furthermore, the expression of ARC and FoxO3a in AS vascular tissue was lower than in the control group, according to RT-qPCR and Western blot analysis (Fig 2I–2K), suggesting their possible roles in the development and progression of AS.

Based on the aforementioned, we hypothesize that miR-150-5p targets FoxO3a to influence macrophage pyroptosis in AS; however, more investigation is required to fully understand its precise mechanism.

### Establishment of cell pyroptosis model

In order to better simulate the onset and growth environment of AS, this study established a model of AS accompanied by cell pyroptosis. According to our established ox-LDL stimulated RAW264.7 pyroptosis model [28], CCK-8 and LDH assays were utilized to identify the survival of cells and rupture of membranes, respectively. S2A and S2B Fig show that as the concentration of ox-LDL increases (0–200 mg/L), cell viability gradually increases gradually decreasing, while LDH release increases accordingly. Additionally, ox-LDL (0–200 mg/L) induced pyroptosis in a dose-dependent manner. S2C–S2F Fig shows that as the solubility of ox-LDL increases, the expression levels of pyroptosis related proteins also increase, namely NLRP3, Caspase-1 (P20), and GSDMD-N protein levels significantly increase, and ox-LDL increases the levels of IL-18 and IL-1 β in the culture medium. The experimental results confirmed that ox-LDL activated NLRP3 inflammasome and GSDMD-N, leading to the release of IL-18 and IL-1 β. Given that 200 mg/L ox-LDL caused approximately 50% reduction in cell viability, this concentration was used in subsequent experiments, confirming the stability of the injury model.

### MiR‑150‑5p inhibitor alleviates ox-LDL‑stimulated injury among RAW264.7

**Fig 3:**
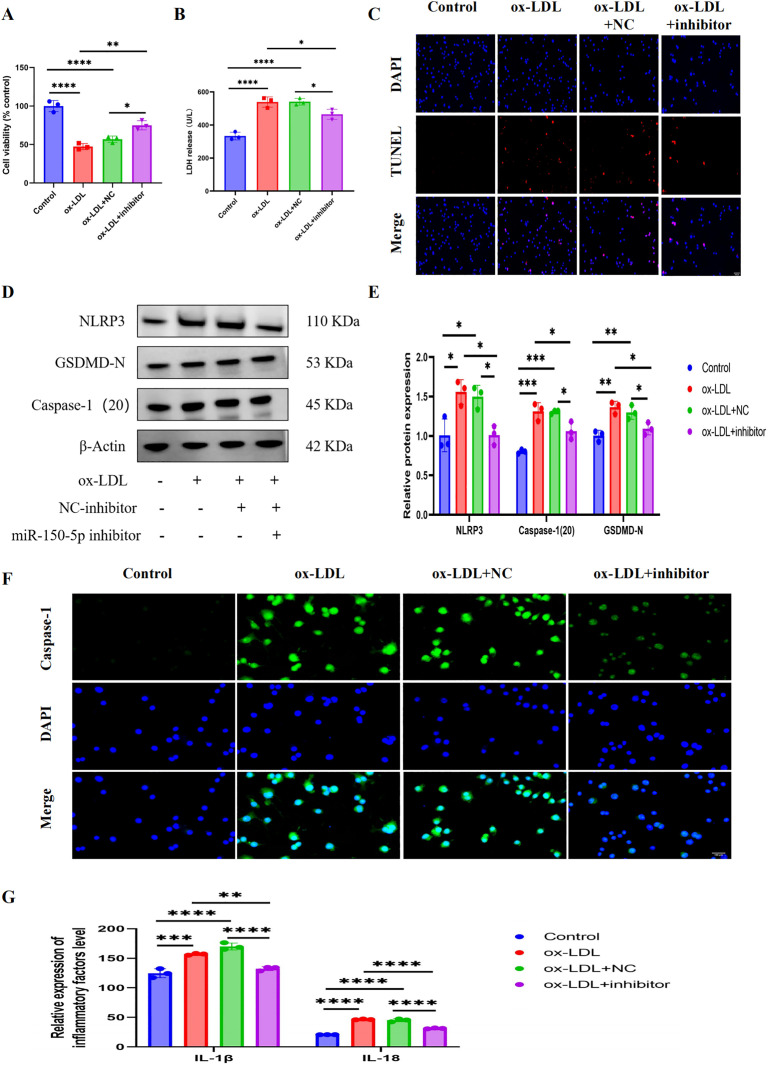
Therapeutic effects of miR-150-5p inhibition on ox-LDL-induced pyroptosis in RAW264.7 macrophages. (A) Cell viability measured by CCK-8 assay. (B) Cytotoxicity assessed by LDH release into culture media. (C) TUNEL staining showing DNA fragmentation (red) with DAPI counterstain (blue). Scale bar: 100 μm. (D-E) Western blot analysis of pyroptosis-related proteins (cleaved GSDMD, NLRP3, ASC). (F) Immunofluorescence detection of Caspase-1 activation (green) with nuclear counterstain (DAPI, blue). Scale bar: 100 μm. (G) Secreted inflammatory cytokines (IL-1β, IL-18) quantified by ELISA. Data are described with mean±SD of at least 3 diferent experiments. ns = not signifcant; *P < 0.05; **P < 0.01; ***P < 0.001; ****P < 0.0001.

Ox-LDL treatment reduced cell survival and increased LDH leakage, but transfection with miR-150-5p inhibitors weakened these effects (Fig 3A and 3B). To evaluate the protective effect of miR-150-5p inhibitors in RAW264.7 cells stimulated by ox-LDL, TUNEL assay was used to assess DNA damage. Ox-LDL induced significant DNA damage in RAW264.7 cells, while miR-150-5p inhibitor transfection significantly reduced this damage (Fig 3C), highlighting the protective effect of miR-150-5p inhibition on Ox-LDL induced macrophage damage.

### MiR-150-5p inhibitor restricts RAW264.7 pyroptosis in ox-LDL attack

This study indicates that ox-LDL induced cytotoxicity significantly activates the pyroptosis pathway, with increased expression of pyroptosis related proteins NLRP3, Caspase-1 (P20), and GSDMD-N, as well as increased secretion of IL-18 and IL-1 β (S2 Fig). In contrast, transfection with miR-150-5p inhibitors significantly reversed the oxLDL-induced upregulation of these signaling molecules (Fig 3D and 3E). To further investigate Caspase-1-dependent pyroptosis, a fluorescence assay confirmed that miR-150-5p inhibitors reduced the accumulation of Caspase-1 fluorescence in oxLDL-treated RAW264.7 cells (Fig 3F). Additionally, miR-150-5p inhibitors suppressed the oxLDL-induced secretion of IL-18 and IL-1β (Fig 3G). These findings suggest that miR-150-5p inhibitors may exert protective effects by inhibiting the activation of NLRP3 inflammasome, GSDMD cleavage, and the release of pro-inflammatory cytokines IL-18 and IL-1 β.

### MiR-150-5p inhibitor promotes activation of FoxO3a/ARC axis

**Fig 4:**
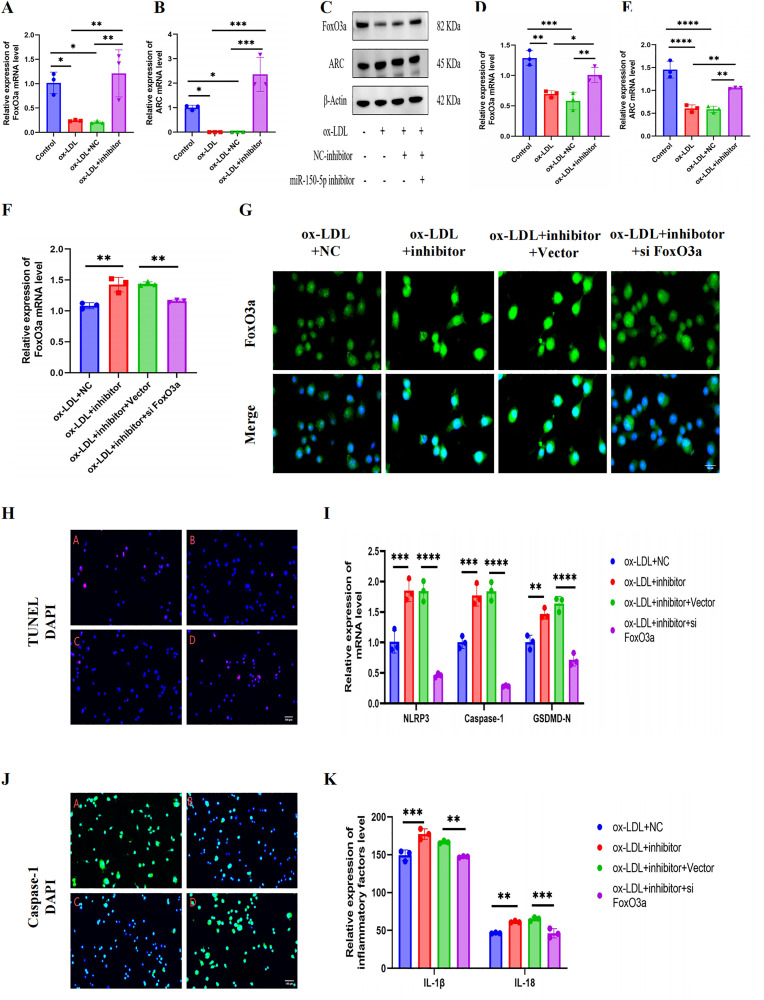
FoxO3a-dependent mechanism of miR-150-5p in regulating pyroptosis in ox-LDL-stimulated RAW264.7 macrophages. (A-E) FoxO3a and ARC expression analyzed by (A-C) RT-qPCR and (D-E) Western blot. (F) FoxO3a mRNA levels quantified by RT-qPCR. (G) Immunofluorescence localization of FoxO3a (green) with DAPI nuclear counterstain (blue). Scale bar: 100 μm. (H) TUNEL assay detecting DNA fragmentation (red) with DAPI counterstain (blue). Scale bar: 100 μm. (I) RT-qPCR analysis of pyroptosis-related genes (NLRP3, ASC, Caspase-1). (J) Immunofluorescence visualization of Caspase-1 activation (green) with DAPI (blue). Scale bar: 100 μm. (K) Secreted IL-1β and IL-18 levels measured by ELISA. A: ox-LDL + NC; B: ox-LDL+inhibitor; C: ox-LDL+inhibitor+Vector; D: ox-LDL+inhibotor+si FoxO3a. Data are described with mean±SD of at least 3 diferent experiments. ns = not signifcant; *P < 0.05; **P < 0.01; ***P < 0.001; ****P < 0.0001.

Bioinformatics analysis revealed that FoxO3a and ARC are downstream targets of miR-150-5p. RT-qPCR and Western blot results showed that the increase in miR-150-5p levels is related to the activation of the FoxO3a/ARC axis. Compared with the control group, ox-LDL treatment resulted in a slight decrease in FoxO3a and ARC protein and mRNA expression. It is worth noting that transfection with miR-150-5p inhibitor remarkable upregulated the expression of FoxO3a and ARC proteins in ox-LDL treated cells (Fig 4A–4E). The results indicate that miR-150-5p may inhibit pyroptosis by activating the FoxO3a/ARC axis.

We conducted gain-of-functional and rescue assays to further investigate the role of miR-150-5p in the FoxO3a/ARC axis of macrophages exposed to ox-LDL. We found that transfection with miR-150-5p inhibitor increased the expression of FoxO3a protein. However, when miR-150-5p inhibitor was co-transfected with FoxO3a siRNA, this expression was reduced (Fig 4F). Immunofluorescence staining also showed that compared with the miR-NC group, miR-150-5p inhibitors increased the expression of FoxO3a, which was alleviated by co-inhibition of miR-150-5p and FoxO3a (Fig 4G). These findings collectively suggest that miR-150-5p can regulate the FoxO3a/ARC axis in ox-LDL-stimulated macrophages, where miR-150-5p inhibition activates the axis, potentially mitigating pyroptosis and inflammation.

### siRNA-mediated FoxO3a knockdown abolishes the anti-pyroptotic effect of miR-150-5p inhibition

We conducted further experiments to determine whether miR-150-5p promotes NLRP3 inflammasome mediated pyroptosis through the FoxO3a/ARC pathway. The results showed that transfection of FoxO3a siRNA eliminated the protective effect of miR-150-5p inhibitor on oxLDL induced cellular DNA damage (Fig 4H). The protein expression levels of pyroptosis related proteins, namely NLRP3, Caspase-1 (P20), and GSDMD-N, increased (Fig 4I), the accumulation of Caspase-1 fluorescence increased (Fig 4J), and the secretion of IL-18 and IL-1 β increased (Fig 4K), which confirms this. These observations suggest that the protective effect of miR-150-5p inhibitors on pyroptosis may be mediated through the FoxO3a/ARC axis.

Based on the above, we hypothesize that when activated by miR-150-5p, the FoxO3a/ARC axis acts as an upstream signaling pathway, promoting NLRP3 mediated pyroptosis in RAW264.7 cells exposed to ox-LDL. This suggests a sequential regulatory mechanism where miR-150-5p influences pyroptosis through modulating the FoxO3a/ARC axis, which in turn affects NLRP3 inflammasome activation.

### MiR-150-5p inhibits FoxO3a/ARC axis and promotes Apoe ^-/-^ mouse NLRP3 inflammatory/GDMD mediated pyroptosis

**Fig 5:**
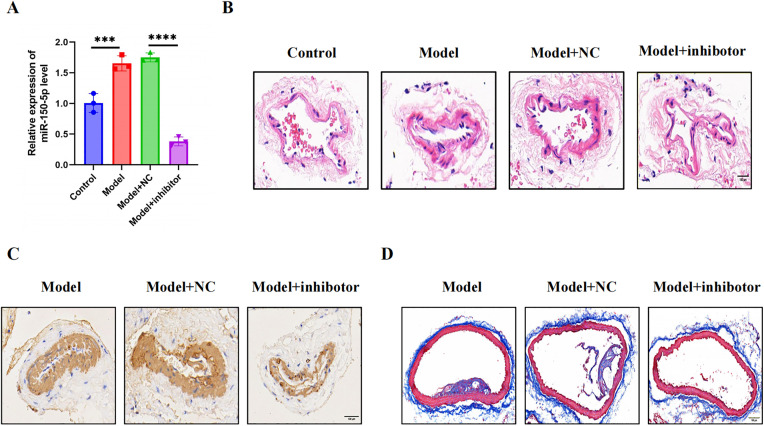
Therapeutic effects of miR-150-5p inhibition on atherosclerotic lesion development. (A) RT-qPCR analysis demonstrating significant knockdown of miR-150-5p in vascular tissues following inhibitor treatment. (B) Atherosclerotic plaque morphology in aortic root sections (HE staining). Scale bar: 100 μm. (C) Vascular smooth muscle cell content assessed by α-SMA immunohistochemistry. Scale bar: 100 μm. (D) Fibrous cap collagen deposition visualized by Masson’s trichrome staining. Although the typical three leaflet structures of the aortic valve can not be fully displayed on this slice plane, this image clearly shows the load of atherosclerotic plaque and its relationship with adjacent cardiac structures. Scale bar: 100 μm. Data are described with mean±SD of at least 3 diferent experiments. ns = not signifcant; *P < 0.05; **P < 0.01; ***P < 0.001; ****P < 0.0001.

An inhibitor was used to downregulate miR-150-5p in mouse vascular tissue in order to examine its function in an AS mice model. RT-qPCR confirmed significant miR-150-5p reduction in inhibitor-treated AS mice compared to controls (Fig 5A).

**Fig 6:**
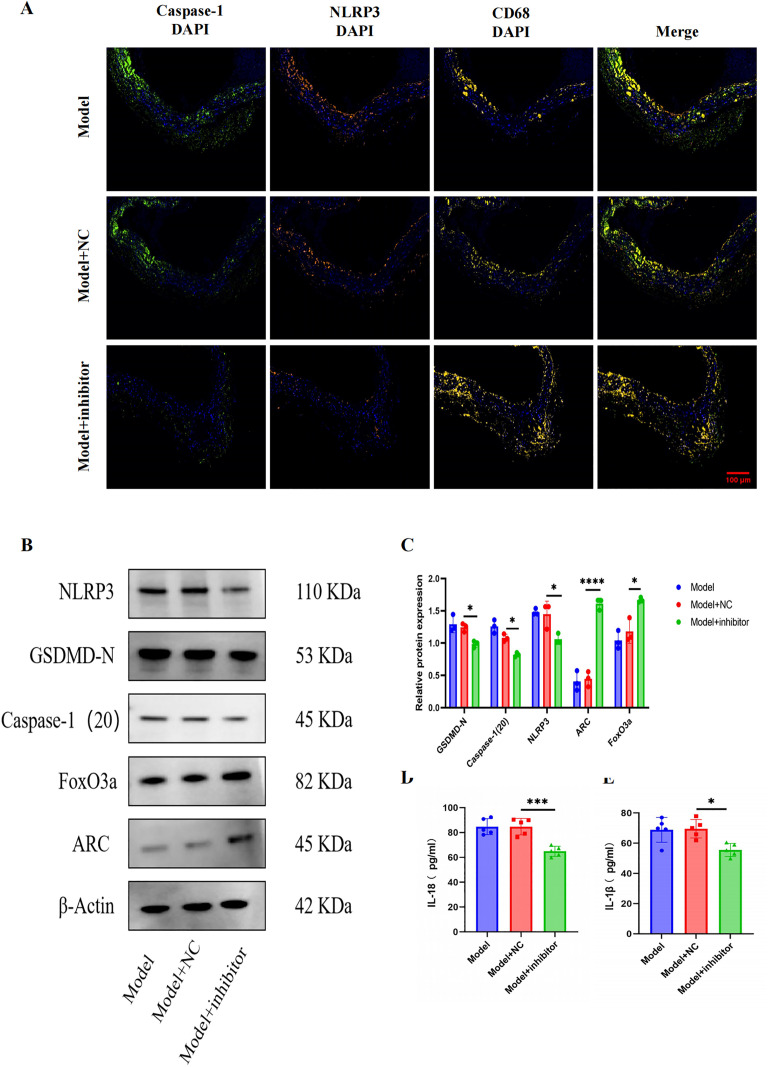
miR-150-5p inhibition modulates pyroptosis and FoxO3a/ARC signaling in ApoE^-/-^ mice. (A) Triple immunofluorescence staining of aortic roots showing colocalization of CD68 + macrophages (yellow) with NLRP3 (orange) and Caspase-1 (green). DAPI (blue) stains nuclei. Scale bar: 100 μm. (B) Western blot analysis of pyroptosis-related proteins (NLRP3, Caspase-1, GSDMD) and FoxO3a/ARC pathway components in aortic arches. (C) Serum concentrations of IL-1β and IL-18 measured by ELISA. Data are described with mean±SD of at least 3 diferent experiments. ns = not signifcant; *P < 0.05; **P < 0.01; ***P < 0.001; ****P < 0.0001.

H&E staining showed thinner vascular walls, reduced lipid deposition, and no thrombus formation in inhibitor-treated mice, contrasting with vascular thickening and thrombosis in PBS/NC groups (Fig 5B). Compared with PBS and NC groups, 150-5p inhibitor significantly reduced the atherosclerotic lesion area in Apoe ^-/-^mice’s aortic root plaque, and increased the content of collagen and fibrin (Fig 5B–5D). Fig 6 illustrates how miR-150-5p inhibitors drastically decreased the expression of NLRP3, Caspase-1, and GSDMD-N in comparison to the model group. They also decreased the levels of serum IL-18 and IL-1 β. Additionally, it upregulated FoxO3a and ARC in aortic arches. These findings imply that miR-150-5p may promote macrophage pyroptosis and amage to DNA in lesions related to atherosclerosis through the FoxO3a/ARC axis.

## Discussion

AS has a significant role in the occurrence and progression of cardiovascular diseases, and is among the primary causes of death for the world’s aging population. To prevent and treat cardiovascular disorders, it is essential to investigate the molecular mechanisms underlying AS development. Exosomes are closely related to the control of various physiological processes and play important roles in different stages of AS formation. In recent years, research has found that pyroptosis is the main cause of macrophage death in AS plaques, and is closely related to plaque instability. There are limited findings on how exosome-regulated macrophage pyroptosis affects AS, despite studies demonstrating that exosome miRNAs contribute to macrophage pyroptosis in a variety of disorders. The complexity of macrophage pyroptosis in AS plaque erosion and rupture is still not fully elucidated; Pyroptosis is a key mechanism in atherosclerosis, where Caspase-1 activation drives ox-LDL-induced macrophage death, leading to the development of unstable plaques [9]. Specifically, we established that ox-LDL induces macrophage pyroptosis in a dose-dependent manner, providing a direct connection between a major atherogenic stimulus and cellular instability. Moreover, we revealed a central regulatory role of the miR-150-5p/FoxO3a axis in this process. Inhibition of miR-150-5p significantly suppressed pyroptosis and reduced the secretion of IL-1β and IL-18, whereas siRNA-mediated knockdown of FoxO3a reversed this protective effect. These results not only validate FoxO3a as a functional target of miR-150-5p, but also uncover a novel pathway regulating inflammatory cell death in AS. Taken together, our findings suggest that targeting miR-150-5p may represent a promising therapeutic strategy to attenuate pyroptosis-related plaque vulnerability and promote plaque stabilization.

We systematically compared the miRNA sequences in the vascular tissues of AS and normal control mice in this investigation. The findings suggested that miR-150-5p might be a pathogenic cause of vascular tissues since miR-150-5p expression was significantly higher in the vascular tissues of mice in the AS group. Furthermore, bioinformatics investigation revealed that FoxO3a is miR-150–5 p’s downstream target. FoxO3a and ARC expression levels decreased in the AS group, according to the findings of the RT-qPCR and Western blot investigations. Thus, FoxO3a and ARC are potential AS therapeutic targets, with the FoxO pathway implicated in disease mechanisms and treatment strategies.

Pyroptosis, also referred to as cell proinflammatory necrosis, constitutes a pro-inflammatory cell death triggered by Caspase-1 family and GSDMD protein activation. During the process of pyroptosis, intracellular substances and a large amount of pro-inflammatory mediators are released, IL −1 β, IL-18, etc., which are closely related to inflammation [5,6]. Pyroptosis is an important innate immune mechanism involved in viral infections, neurological disorders, and atherosclerosis. Emerging evidence indicates that pyroptosis promotes atherosclerotic progression through inflammatory mediator release and directly correlates with plaque destabilization [29]. Understanding pyroptosis’ molecular mechanisms in atherosclerotic initiation and progression is essential for deciphering disease pathogenesis and discovering new therapeutic interventions.

Exosomes, created by cells damaged in pathologic locations may transport microRNA, mRNA, and protein-based transcription factors to particular cells, modifying their phenotype. In recent years, research on macrophage pyroptosis induced by exosomes miRNAs has been reported in various diseases, such as sepsis related acute lung injury where neutrophil extracellular vesicle miR-30d-5p induces polarization of M1 macrophages and triggers macrophage pyroptosis [30], plasma derived exosomes trigger NLRP3 dependent pyroptosis of alveolar macrophages, leading to pancreatitis related lung injury [31], and so on. However, there are few reports on their research in AS. Using miR-150-5p antagonist administration in both in vivo and in vitro models with proper controls, we demonstrated that the downregulation of miR-150-5p in tissue exosomes contributes to atherosclerotic pathogenesis. According to the findings, there was no discernible thrombus formation, lipid deposition was decreased, and the arterial wall was thinner in the miR-150-5p inhibitor treatment group. This suggests that the increase of miR-150-5p is a significant contributing factor for AS. The miR-150-5p inhibitor delays plaque formation by regulating cell pyroptosis. The evaluation of aortic root slices revealed significant atherosclerotic plaque formation. The focus of this histological analysis is plaque characteristics and overall load. Although the orientation of some slices failed to clearly show all three valve lobes at the same time, the observed images clearly showed the load of atherosclerotic plaque and its relationship with adjacent cardiac structures. The severity of plaque observed in these slices is enough to support our conclusions.

By binding to the 3’UTR of mRNAs, miRNAs function as upstream regulators that can post-transcriptionally suppress mRNA expression and modulate the translational activity and biological functions of target genes [32]. Research shows that miRNAs are closely related to cardiovascular disease [33]. We examined FoxO3a, the downstream targets of miR-150-5p, to learn more regarding how miR-150-5p inhibitors work. We were able to confirm that FoxO3a is indeed a miR-150-5p target gene using dual-luciferase labeling. Rescue tests were carried out to determine if miR-150-5p functions as an upstream molecule of FoxO3a that alters macrophage biological processes. It has been suggested that miR-150-5p may target FoxO3a as a direct binding partner since co-transfection of FoxO3a siRNA and miR-150-5p inhibitor may mitigate the inhibitory effect of miR-150-5p inhibitors on macrophage pyroptosis.

To further explore the role of FoxO3a in AS, we delved into another important key gene, ARC. Our experiments demonstrated that upon ox-LDL stimulation, miR-150-5p expression increased, while FoxO3a and ARC expression decreased. In contrast, miR-150-5p inhibition upregulated FoxO3a and ARC expression. These results imply that the FoxO3a-ARC axis inside the FoxO pathway is how miR-150-5p functions. The proposed mechanism involves reducing macrophage pyroptosis, thereby decreasing the formation of AS plaques.

Finally, while our study demonstrates a strong association between the miR-150-5p/FoxO3a axis and macrophage pyroptosis both in vitro and in vivo, we acknowledge certain limitations. The mechanistic insights, particularly concerning the cell-autonomous role of macrophage FoxO3a, were primarily derived from siRNA knockdown experiments in cultured cells. To unequivocally confirm that the anti-pyroptotic effects of miR-150-5p inhibition are mediated specifically through macrophage FoxO3a in vivo, future studies using macrophage-specific knockout models are essential. This approach would also rule out potential contributions from other cell types. Furthermore, our work does not fully address the significant heterogeneity within the monocyte-macrophage lineage. It is highly plausible that the observed anti-pyroptotic effects are more pronounced in a specific inflammatory subset, such as pro-inflammatory or foam-cell macrophages. This cellular heterogeneity carries profound therapeutic implications, suggesting that the efficacy of miR-150-5p-targeted intervention may depend critically on the abundance of susceptible subsets in the plaque microenvironment. Thus, identifying the precise sensitive macrophage subpopulation and defining the optimal therapeutic window for intervention represent critical and compelling priorities for future investigation.

### Conclusion

In conclusion, our research primarily revealed that FoxO3a upregulation could prevent macrophage pyroptosis, which is stimulated by miR-150-5p. These results suggested that FoxO3a, ARC, and miR-150-5p regulated the onset and course of macrophage pyroptosis, which may offer a new and promising therapeutic target for AS and improve a close understanding of the underlying mechanisms.

## Supporting information

S1 Fig Metabolic and molecular effects of high-fat diet (HFD). (A-B) Body weight changes. (C-D) Atherosclerotic plaque morphology (HE staining, 20×). (E-F) Serum inflammatory markers. (G-H) Pyroptosis-related protein levels. (I) Pyroptosis-related gene expression. Data are described with mean±SD of at least 3 diferent experiments. ns = not signifcant; *P < 0.05; **P < 0.01; ***P < 0.001.
(TIF)

S2 Fig ox-LDL stimulates cytotoxicity and pyroptosis among RAW264.7. RAW264.7 were incubated with oxLDL (0–200 mg/L) for 24 h. CCK8 assay (A) and LDH assay (B) showing cell viability and LDH activity in media. (C, D) WB assays showing the protein levels of pyroptosis-related makers. ELISA assays for IL-18 (E) and IL-1β (F) in media. Data are described with mean±SD of at least 3 diferent experiments. Data are described with mean±SD of at least 3 diferent experiments. ns = not signifcant; *P < 0.05; **P < 0.01; ***P < 0.001; ****P < 0.0001.
(TIF)

S1 File S3_Raw_Images.pdf. (PDF)

S2 File S4 Minimal Data Set. Pzfx. (PZFX)

## Abbreviation

AS: Atherosclerosis
ARC: Apoptosis inhibitors containing caspase recruitment domains
BMSCs: Bone marrow mesenchymal stem cells
BP: Biological processes
CVD: Cardiovascular disease
CC: Cellular components
ELISA: Enzyme-linked immunosorbent assay
FoxO: Forkhead box O
GO: Gene Ontology
GDP: Gross Domestic Product
IL: Interleukin
KEGG: Kyoto Encyclopedia of Genes and Genomes
LDH: Lactic dehydrogenase
nFCM: Nanoflow cytometry
NTA: Nanoparticle Tracking Analysis
MF: Molecular functions
OGD/R: Oxygen glucose deprivation/reperfusion
PBS: Phosphate buffered saline
RT-qPCR: Real-time quantitative reverse-transcription PCR
SMC: smooth muscle cell
TEM: Transmission electron microscope
TNF-α: Tumor necrosis factor- α
VSMCs: Vascular smooth muscle cells
WB: Western bloting

## References

[1] He X, Fan X, Bai B, Lu N, Zhang S, Zhang L. Pyroptosis is a critical immune-inflammatory response involved in atherosclerosis. Pharmacol Res. 2021;165:105447. doi: 10.1016/j.phrs.2021.105447 33516832

[2] Arnett DK, Blumenthal RS, Albert MA, Buroker AB, Goldberger ZD, Hahn EJ, et al. 2019 ACC/AHA guideline on the primary prevention of cardiovascular disease: executive summary: a report of the American College of Cardiology/American Heart Association Task Force on Clinical Practice Guidelines. Circulation. 2019;140(11):e563–95. doi: 10.1161/CIR.0000000000000677 30879339 PMC8351755

[3] Ma LY, Wu YZ, Chen WW. Key points of the 2018 Chinese cardiovascular disease report. Chin J Hypertens. 2019;27(08):712–6.

[4] Khatana C, Saini NK, Chakrabarti S, Saini V, Sharma A, Saini RV, et al. Mechanistic insights into the oxidized low-density lipoprotein-induced atherosclerosis. Oxid Med Cell Longev. 2020;2020:5245308. doi: 10.1155/2020/5245308 33014272 PMC7512065

[5] Xu J, Jiang Y, Wang J, Shi X, Liu Q, Liu Z, et al. Macrophage endocytosis of high-mobility group box 1 triggers pyroptosis. Cell Death Differ. 2014;21(8):1229–39. doi: 10.1038/cdd.2014.40 24769733 PMC4085529

[6] Byrne BG, Dubuisson JF, Joshi AD, Persson JJ, Swanson MS. Inflammasome components coordinate autophagy and pyroptosis as macrophage responses to infection. mBio. 2013;4(1):e00620-12. doi: 10.1128/mBio.00620-12 PMC3573666 23404401

[7] Wei Y, Lan B, Zheng T, Yang L, Zhang X, Cheng L, et al. GSDME-mediated pyroptosis promotes the progression and associated inflammation of atherosclerosis. Nat Commun. 2023;14(1):929. doi: 10.1038/s41467-023-36614-w 36807553 PMC9938904

[8] Guo W, Huang R, Bian J, Liao Q, You J, Yong X, et al. Salidroside ameliorates macrophages lipid accumulation and atherosclerotic plaque by inhibiting Hif-1α-induced pyroptosis. Biochem Biophys Res Commun. 2025;742:151104. doi: 10.1016/j.bbrc.2024.151104 39642710

[9] Zeng W, Wu D, Sun Y, Suo Y, Yu Q, Zeng M, et al. The selective NLRP3 inhibitor MCC950 hinders atherosclerosis development by attenuating inflammation and pyroptosis in macrophages. Sci Rep. 2021;11(1):19305. doi: 10.1038/s41598-021-98437-3 34588488 PMC8481539

[10] Link W. Introduction to FOXO biology. Methods Mol Biol. 2019;1890:1–9. 30414140 10.1007/978-1-4939-8900-3_1

[11] Jang H, Na Y, Hong K, Lee S, Moon S, Cho M, et al. Synergistic effect of melatonin and ghrelin in preventing cisplatin-induced ovarian damage via regulation of FOXO3a phosphorylation and binding to the p27Kip1 promoter in primordial follicles. J Pineal Res. 2017;63(3):10.1111/jpi.12432. doi: 10.1111/jpi.12432 28658519

[12] Ali T, Rahman SU, Hao Q, Li W, Liu Z, Ali Shah F, et al. Melatonin prevents neuroinflammation and relieves depression by attenuating autophagy impairment through FOXO3a regulation. J Pineal Res. 2020;69(2):e12667. doi: 10.1111/jpi.12667 32375205

[13] Thompson MG, Larson M, Vidrine A, Barrios K, Navarro F, Meyers K. FOXO3-NF-kappaB RelA protein complexes reduce proinflammatory cell signaling and function. J Immunol. 2015;195:5637–47. 26561547 10.4049/jimmunol.1501758 PMC4670825

[14] Liu Z, Xiang P, Zeng S, Weng P, Wen Y, Zhang W, et al. N-Acetylneuraminic acid triggers endothelial pyroptosis and promotes atherosclerosis progression via GLS2-mediated glutaminolysis pathway. Cell Death Discov. 2024;10(1):467. doi: 10.1038/s41420-024-02233-7 39537619 PMC11561128

[15] Bartel DP. MicroRNAs: genomics, biogenesis, mechanism, and function. Cell. 2004;116(2):281–97. 14744438 10.1016/s0092-8674(04)00045-5

[16] Kalayinia S, Arjmand F, Maleki M, Malakootian M, Singh CP. MicroRNAs: roles in cardiovascular development and disease. Cardiovasc Pathol. 2021;50:107296. doi: 10.1016/j.carpath.2020.107296 33022373

[17] Xing W, Li T, Wang Y, Qiang Y, Ai C, Tang H. MiR-33a-5p targets NOMO1 to modulate human cardiomyocyte progenitor cells proliferation and differentiation and apoptosis. J Recept Signal Transduct Res. 2021;41(5):476–87. doi: 10.1080/10799893.2020.1825492 33054489

[18] Zhang Z, Li L, Shi H, Chen B, Li X, Zhang Y, et al. Role of circular RNAs in atherosclerosis through regulation of inflammation, cell proliferation, migration, and apoptosis: focus on atherosclerotic cerebrovascular disease. Medicina (Kaunas). 2023;59(8):1461. doi: 10.3390/medicina59081461 37629751 PMC10456328

[19] Wu F, Wang F, Yang Q, Zhang Y, Cai K, Liu L, et al. Upregulation of miRNA-23a-3p rescues high glucose-induced cell apoptosis and proliferation inhibition in cardiomyocytes. In Vitro Cell Dev Biol Anim. 2020;56(10):866–77. doi: 10.1007/s11626-020-00518-6 33197036 PMC7723946

[20] Wang X, Li X-L, Qin L-J. The lncRNA XIST/miR-150-5p/c-Fos axis regulates sepsis-induced myocardial injury via TXNIP-modulated pyroptosis. Lab Invest. 2021;101(9):1118–29. doi: 10.1038/s41374-021-00607-4 34045679

[21] Bian Y, Cai W, Lu H, Tang S, Yang K, Tan Y. miR-150-5p affects AS plaque with ASMC proliferation and migration by STAT1. Open Med (Wars). 2021;16(1):1642–52. 34761115 10.1515/med-2021-0357 PMC8569285

[22] Yin Q, He M, Huang L, Zhang X, Zhan J, Hu J. lncRNA ZFAS1 promotes ox-LDL induced EndMT through miR-150-5p/Notch3 signaling axis. Microvasc Res. 2021;134:104118. doi: 10.1016/j.mvr.2020.104118 33278458

[23] Zhang M, Li L, Li S. The role of miR-150-5p/SOCS1 pathway in arsenic-induced pyroptosis of LX-2 cells. Biol Trace Elem Res. 2024. 10.1007/s12011-024-04211-7 38689138

[24] Gierasch LM. The journal of biological chemistry: 2016 onward. J Biol Chem. 2016;291(27):15406–7. 27371571 10.1074/jbc.E116.000001 PMC4933200

[25] Li X, Du N, Zhang Q, Li J, Chen X, Liu X, et al. MicroRNA-30d regulates cardiomyocyte pyroptosis by directly targeting foxo3a in diabetic cardiomyopathy. Cell Death Dis. 2014;5(10):e1479. doi: 10.1038/cddis.2014.430 25341033 PMC4237254

[26] Chen R, Yang M. Melatonin inhibits OGD/R-induced H9c2 cardiomyocyte pyroptosis via regulation of MT2/miR-155/FOXO3a/ARC axis. Int Heart J. 2022;63(2):327–37. 35354753 10.1536/ihj.21-571

[27] Calnan DR, Brunet A. The FoxO code. Oncogene. 2008;27(16):2276–88. doi: 10.1038/onc.2008.21 18391970

[28] Wang T, Tian H, Pan T, Yao S, Yu H, Wu Y, et al. Pinocembrin suppresses oxidized low-density lipoprotein-triggered NLRP3 inflammasome/GSDMD-mediated endothelial cell pyroptosis through an Nrf2-dependent signaling pathway. Sci Rep. 2022;12(1):13885. doi: 10.1038/s41598-022-18297-3 35974041 PMC9381505

[29] Xu Y-J, Zheng L, Hu Y-W, Wang Q. Pyroptosis and its relationship to atherosclerosis. Clin Chim Acta. 2018;476:28–37. doi: 10.1016/j.cca.2017.11.005 29129476

[30] Cheng X-W, Wan Y-F, Zhou Q, Wang Y, Zhu H-Q. MicroRNA‑126 inhibits endothelial permeability and apoptosis in apolipoprotein E‑knockout mice fed a high‑fat diet. Mol Med Rep. 2017;16(3):3061–8. doi: 10.3892/mmr.2017.6952 28713948 PMC5548065

[31] Badacz R, Kleczyński P, Legutko J, Żmudka K, Gacoń J, Przewłocki T. Expression of miR-1-3p, miR-16-5p and miR-122-5p as possible risk factors of secondary cardiovascular events. Biomedicines. 2021;9(8):1055. doi: 10.3390/biomedicines9081055 34440258 PMC8391895

[32] Rasko JEJ, Wong JJ-L. Nuclear microRNAs in normal hemopoiesis and cancer. J Hematol Oncol. 2017;10(1):8. doi: 10.1186/s13045-016-0375-x 28057040 PMC5217201

[33] Lee S, Choi E, Cha M-J, Hwang K-C. Looking for pyroptosis-modulating miRNAs as a therapeutic target for improving myocardium survival. Mediators Inflamm. 2015;2015:254871. doi: 10.1155/2015/254871 26491223 PMC4600493

