# Supplementary figures and images for: FoxO3a regulated by miR-150-5p promotes the pyroptosis of macrophages in atherosclerosis
Source: PLoS One. 2025 Oct 17;20(10):e0327075. doi: 10.1371/journal.pone.0327075 (PMC12533854; doi:10.1371/journal.pone.0327075)

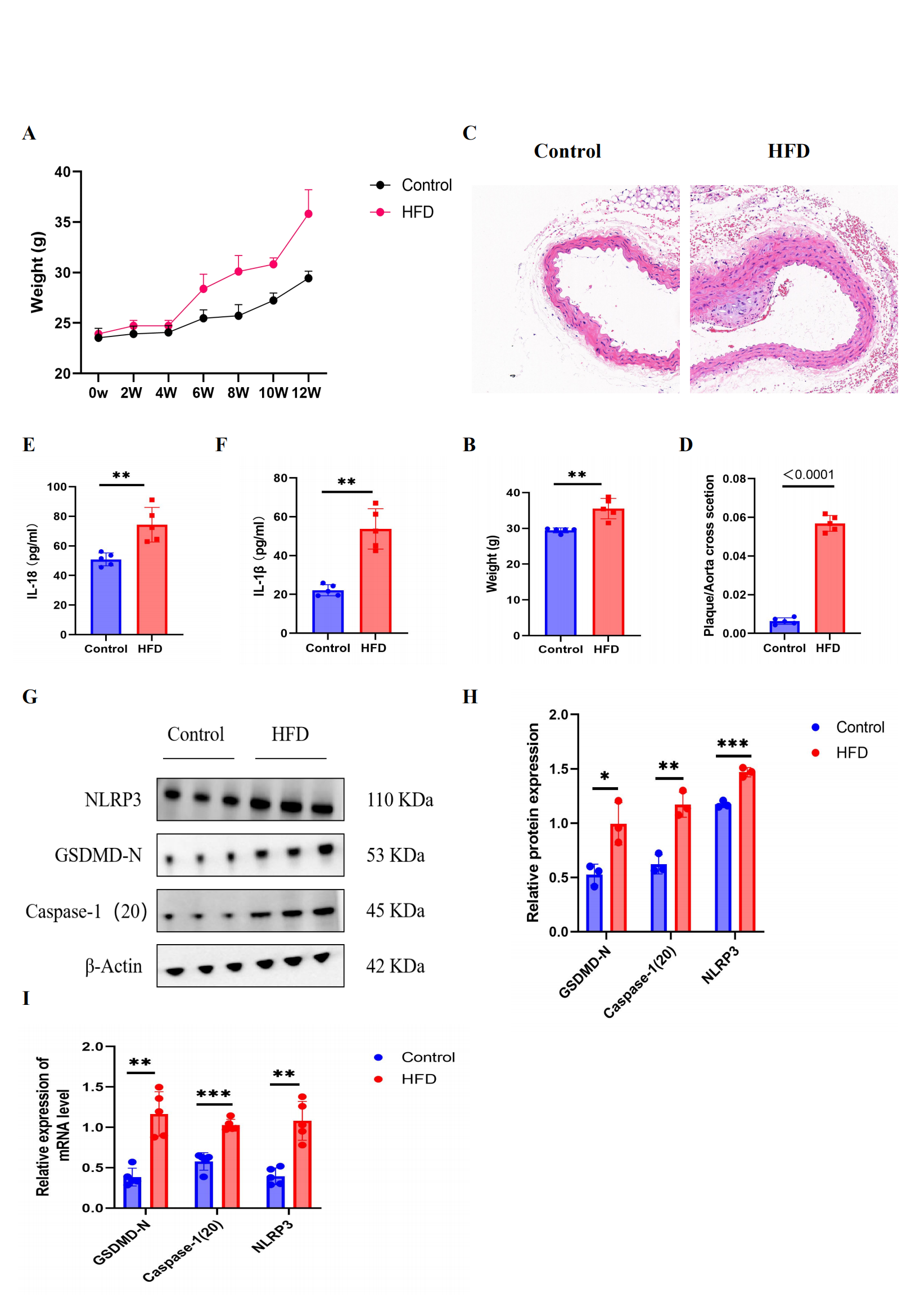

Supplement: S1 Fig — (A-B) Body weight changes. (C-D) Atherosclerotic plaque morphology (HE staining, 20×). (E-F) Serum inflammatory markers. (G-H) Pyroptosis-related protein levels. (I) Pyroptosis-related gene expression. Data are described with mean±SD of at least 3 diferent experiments. ns = not signifcant; *P < 0.05; **P < 0.01; ***P < 0.001. (TIF) [file pone.0327075.s001.tif]

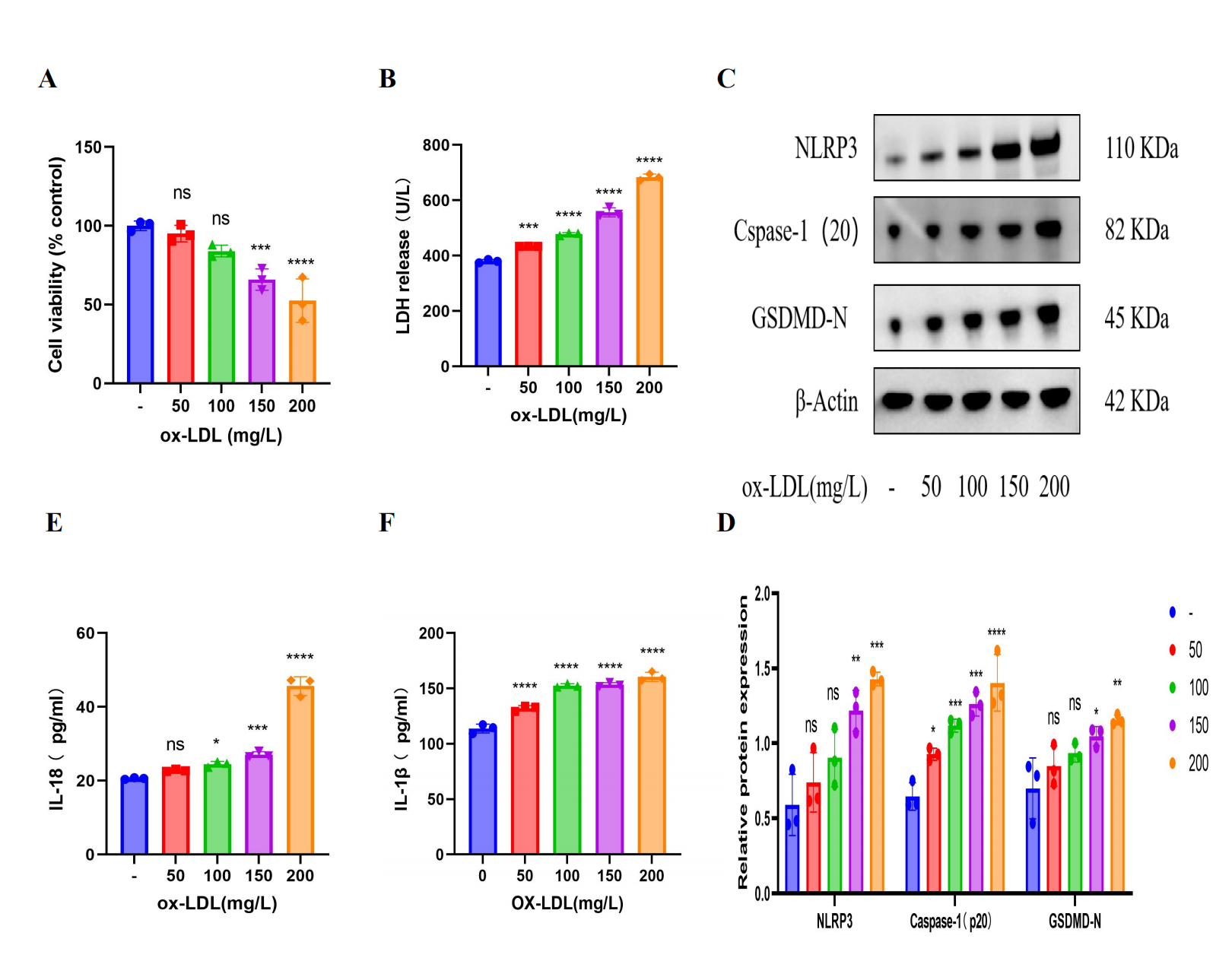

Supplement: S2 Fig — RAW264.7 were incubated with oxLDL (0–200 mg/L) for 24 h. CCK8 assay (A) and LDH assay (B) showing cell viability and LDH activity in media. (C, D) WB assays showing the protein levels of pyroptosis-related makers. ELISA assays for IL-18 (E) and IL-1β (F) in media. Data are described with mean±SD of at least 3 diferent experiments. Data are described with mean±SD of at least 3 diferent experiments. ns = not signifcant; *P < 0.05; **P < 0.01; ***P < 0.001; ****P < 0.0001. (TIF) [file pone.0327075.s002.tif]

Supplementary Figure 1G

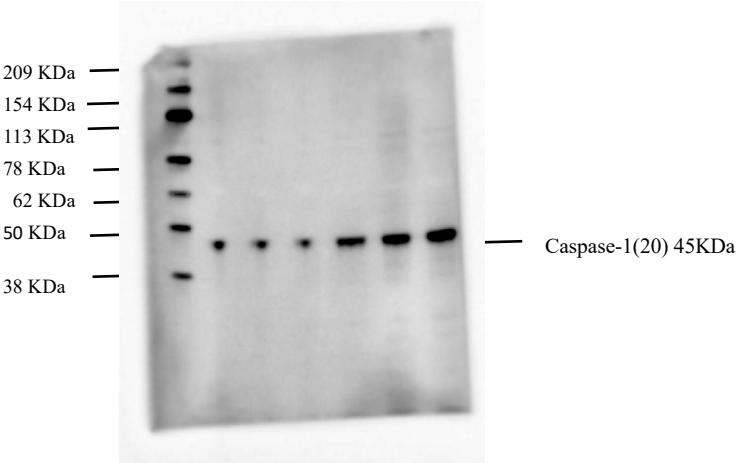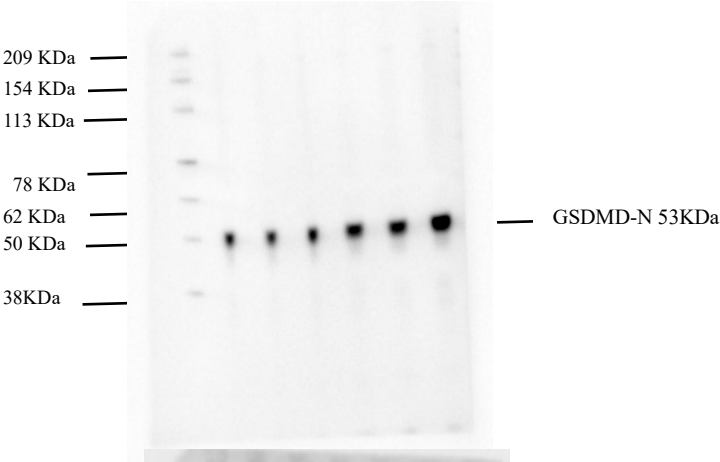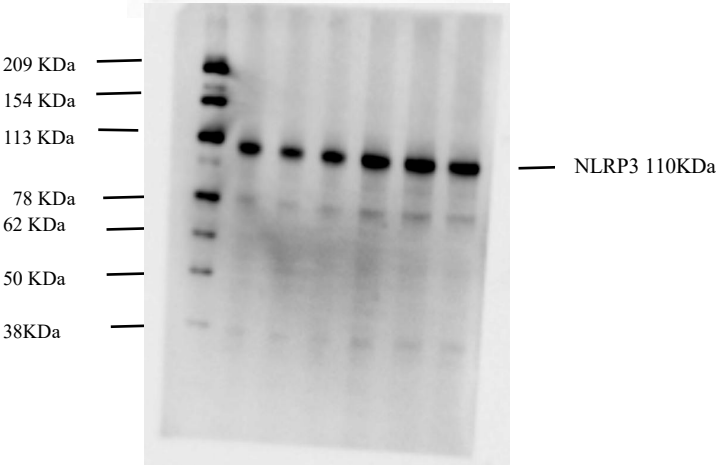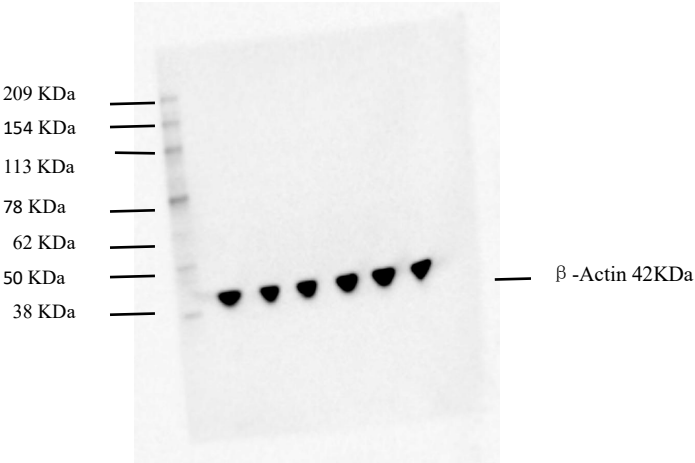

Figure 2J

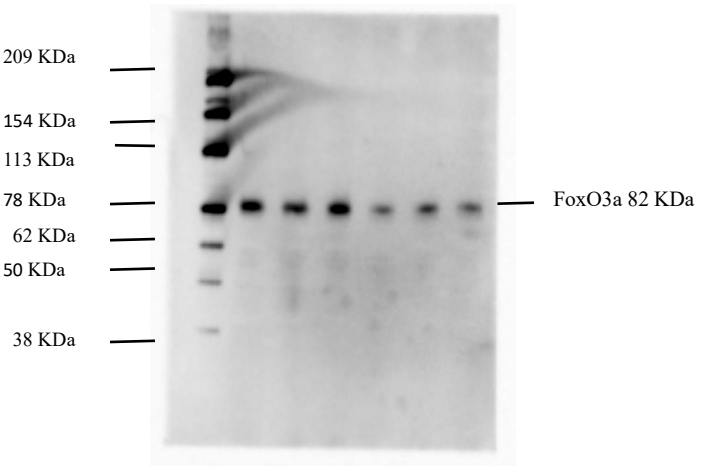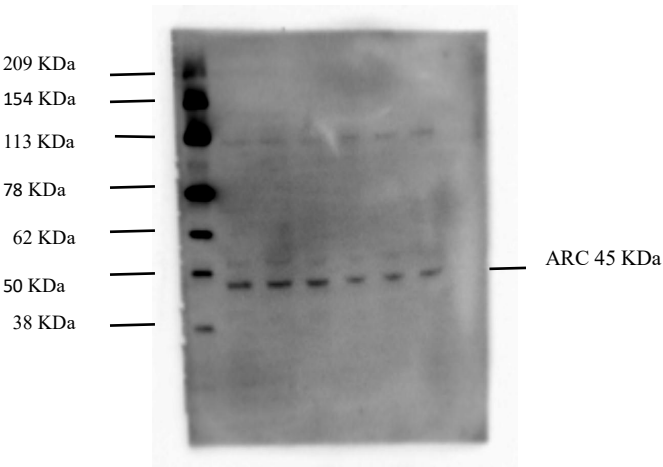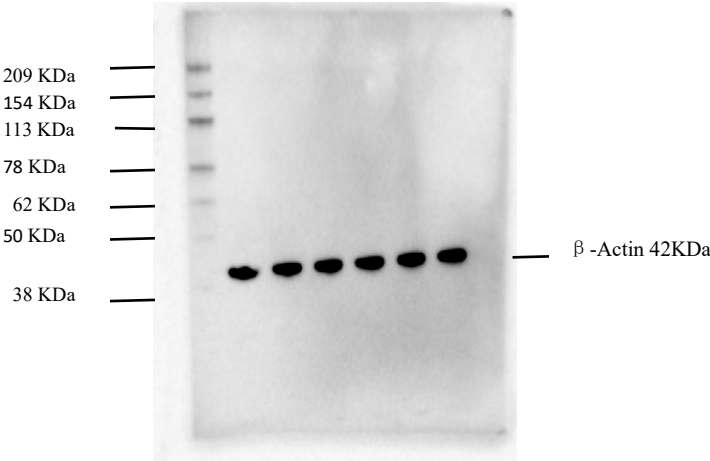

Supplementary Figure 2C

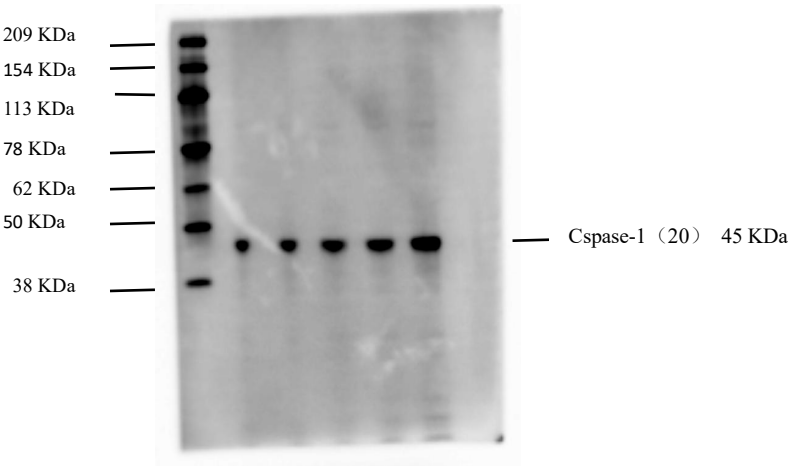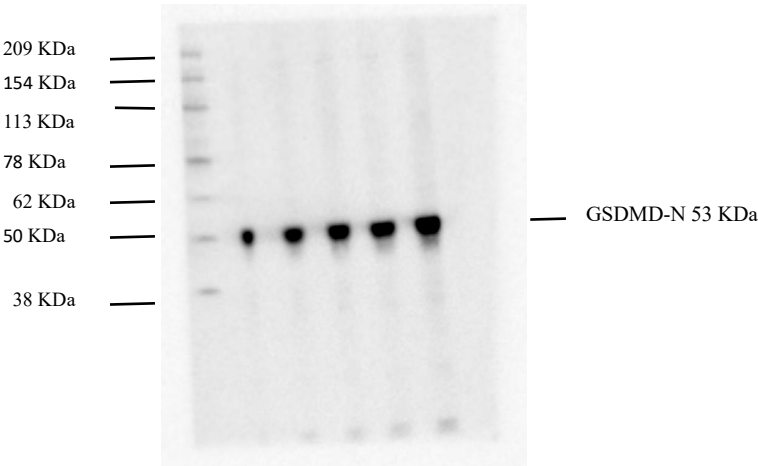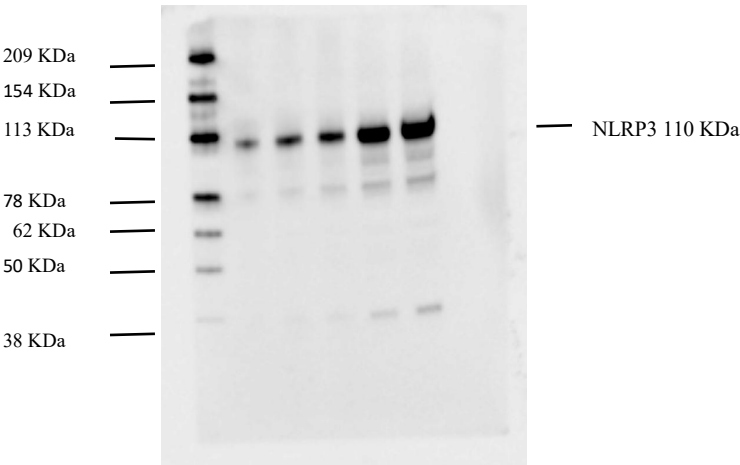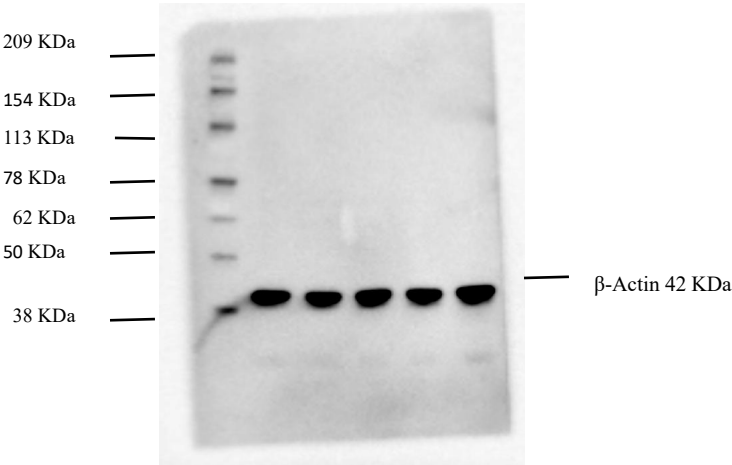

Figure 3D

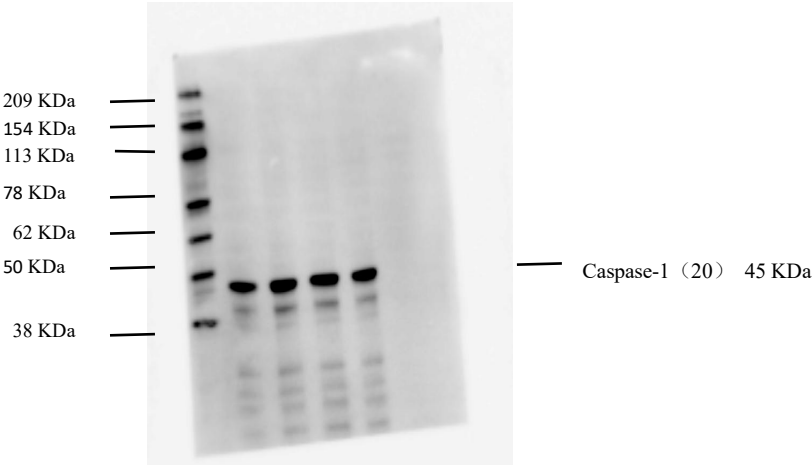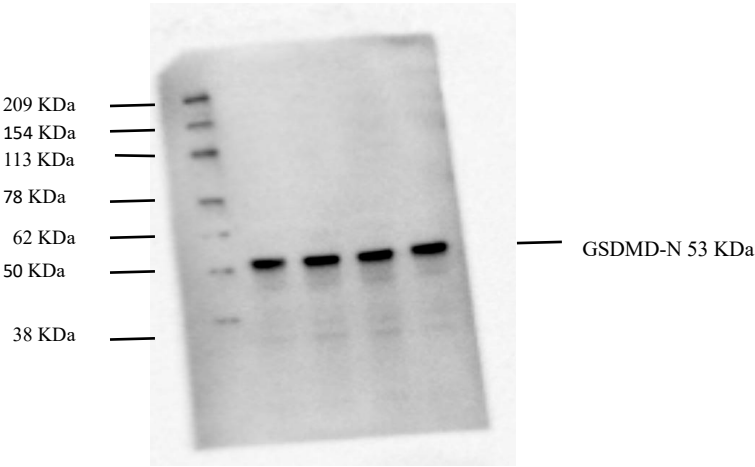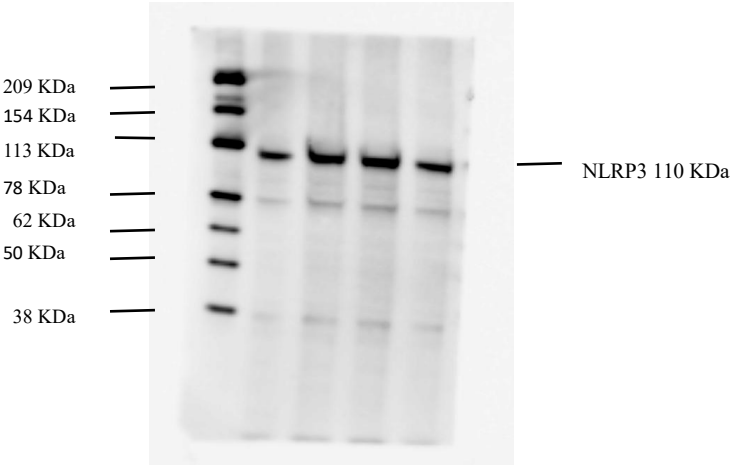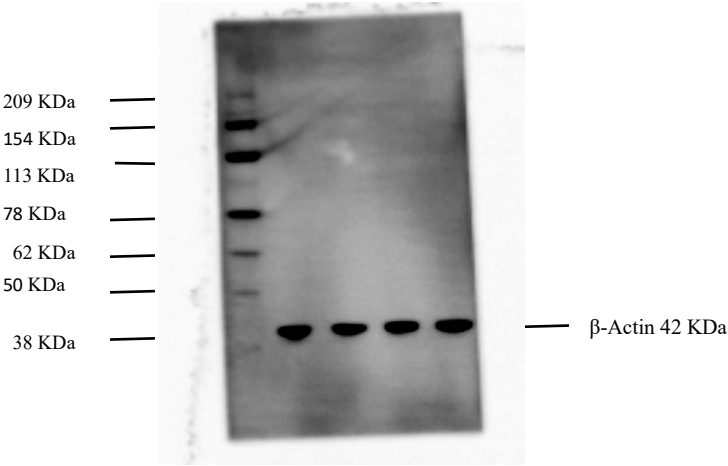

Figure 4C

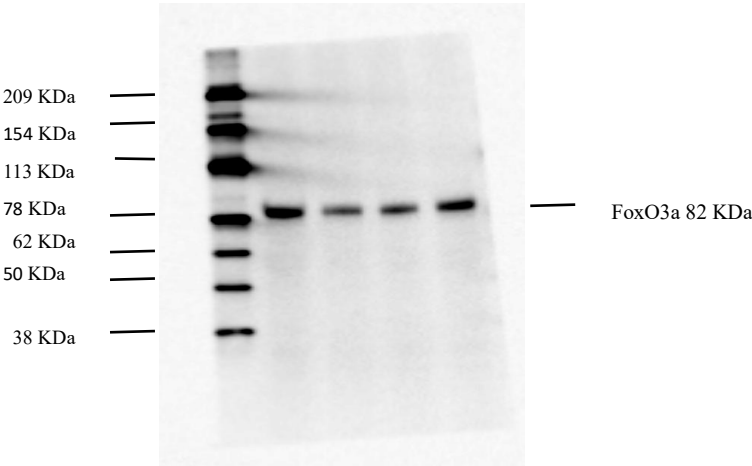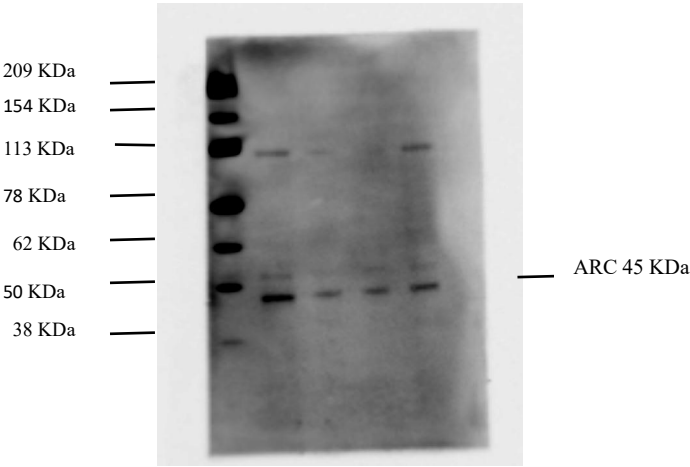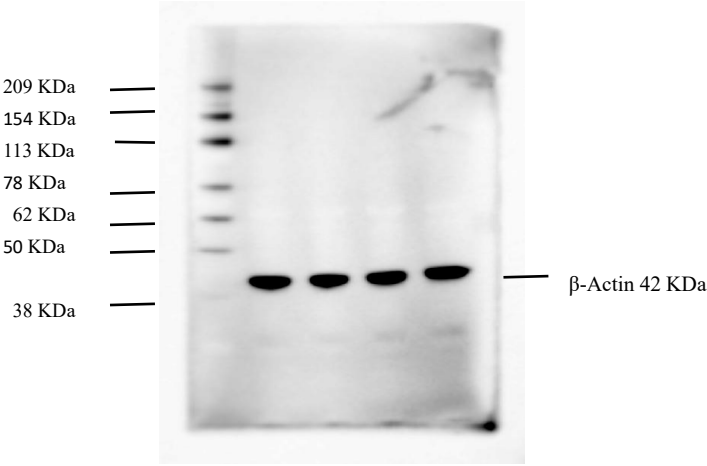

Figure 6B

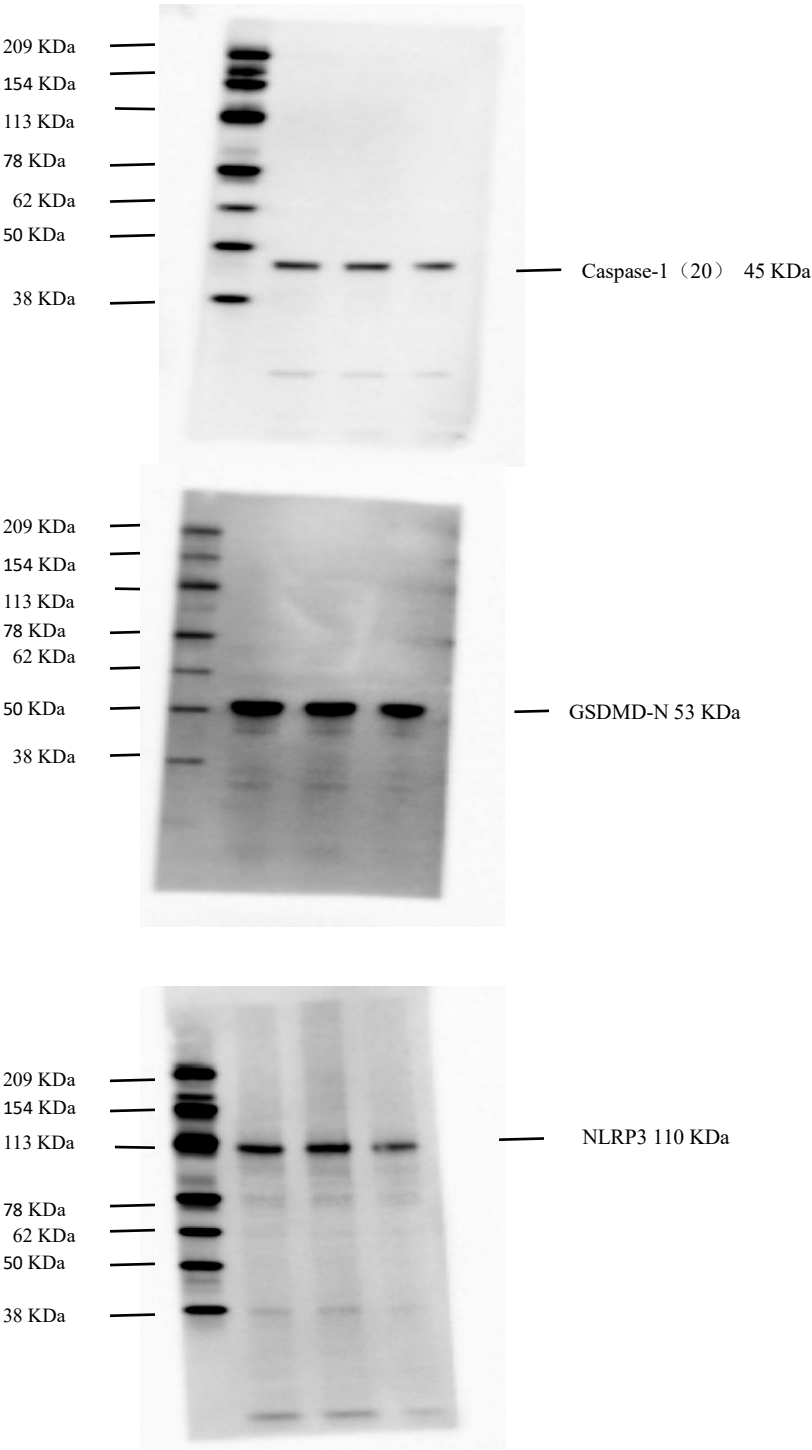

Figure 6B

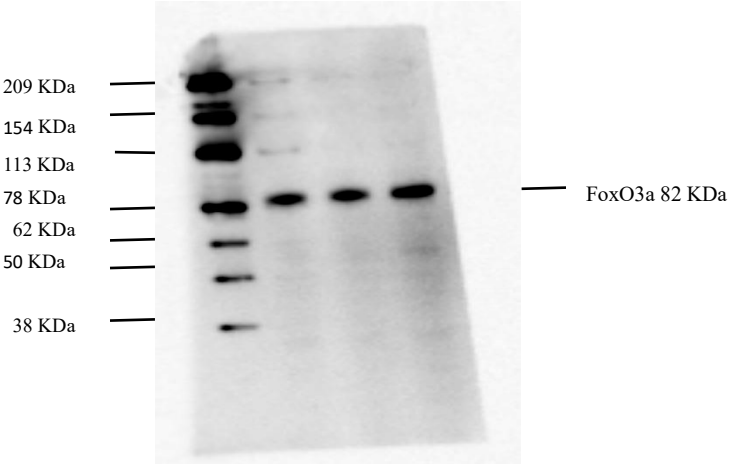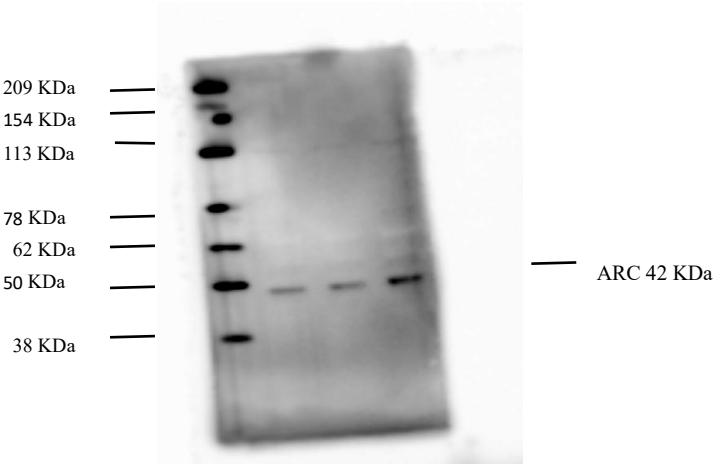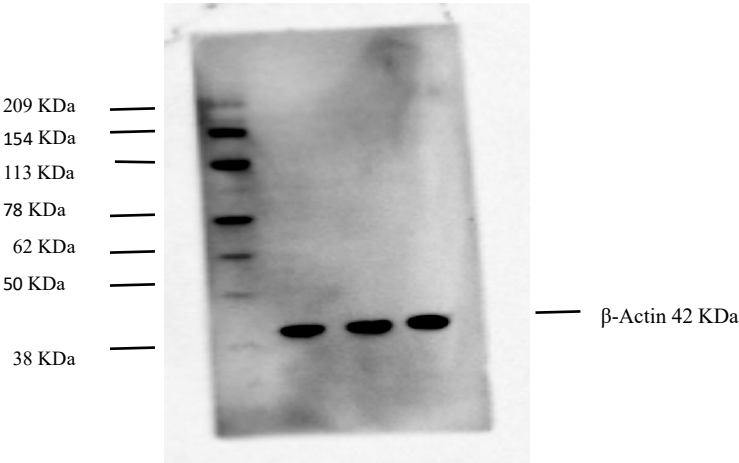

Supplement: S1 File — (PDF) [file pone.0327075.s003.pdf]
